# Instinct Outstunk

**Published:** 1868-10

**Authors:** 


### INSTINCT OUTSTUNK.

"Reasoning at every step he treads,  
Man yet mistakes his way;  
While meaner things, which instinct leads,  
Are rarely known to stray."

So thought, and so sung the poet Christian Cowper; but there are meaner things now than when he lived, and their instinct outstinks any thing then known, unless it be the instinct by which the skunk consecrates its home against the intrusion of impious invaders. Don't you believe it?

Then think of a "Steam Dental Establishment" announcing "a full upper or lower set of teeth for $10 to $15." Imagine a lady caller drawn thither by *steam* power, the magic of the word being the only symptom of steam force used in the place, said patient remarking "you make teeth for ten dollars, I believe." "Well, yes, we sell ready made for ten to fifteen. But your features are a little finer than the average. I fear we have none to fit you, but we'll see." So the patient is seated, and a few worn out "temporary sets" of former patients, not much filthier than something else, are stuck into the mouth. "No, we'll have to take the measure of your mouth, and make a piece to order."

"Are they all the same?"

"Oh, no! made to order they're twenty dollars."

"We'll I reckon I can't do no better."

"Not in this town you can't."

An impression of the mouth with wax, last used in the mouth of a pro——digal, affords a guide in "making a set to order;" the cash is paid, and at least half of the acting parties are satisfied.

Imagine this; and, Q. E. D. There we've touched it!

Chloride of lime? or carbolic acid? Which is best Mr. Health Officer? W.

